# Multisectoral coordination in public health: insights from the 8th EMPHNET regional conference

**DOI:** 10.3389/fpubh.2025.1652755

**Published:** 2025-07-30

**Authors:** Rana AlHamawi, Suhaib Yehya, Faris Lami, Mahmudur Rahman, Muhammad Sartaj, Jonathan E. Suk, Scott F. Dowell, Mohammed Youbi, Heba Mahrous, Vimala Edwin, Abdul Rehman, Mohannad Al Nsour, Magid Al-Gunaid, Yousef Khader, Haitham Bashier

**Affiliations:** ^1^Global Health Development, Eastern Mediterranean Public Health Network, Amman, Jordan; ^2^Texas A and M University, School of Public Health, College Station, TX, United States; ^3^Al Subtain University, and College of Medicine, University of Baghdad, Baghdad, Iraq; ^4^Global Health Development, Eastern Mediterranean Public Health Network, Bangladesh Country Office, Amman, Jordan; ^5^UK Health Security Agency, Belfast, United Kingdom; ^6^European Centre for Disease Prevention and Control, Solna, Sweden; ^7^World Health Organization, Regional Office for the Eastern Mediterranean, Geneva, Switzerland; ^8^Ministry of Health, Rabat, Morocco; ^9^World Health Organization, Regional Office for the Eastern Mediterranean, Cairo, Egypt; ^10^RAK Medical and Health Sciences University, Ras Al-Khaimah, United Arab Emirates; ^11^Department of Public Health, Jordan University of Science and Technology, Irbid, Jordan

**Keywords:** public health, multisectoral coordination, coordination, Eastern Mediterranean Region, collaboration, conference

## Abstract

The concept of multisectoral coordination has been comprehensively described in multiple internationally recognized frameworks. However, the institutionalization of the concept is highly complex and requires further exploration. During the 8th Biennial Regional Conference of the Eastern Mediterranean Public Health Network (EMPHNET), a roundtable session brought together a panel of global and regional experts in multisectoral coordination to share knowledge and expertise, regarding the concept of multisectoral coordination, and the implementation and formalization of coordination mechanisms within national structures, particularly in the Eastern Mediterranean Region (EMR). Discussions also addressed the challenges associated with effective multisectoral coordination, along with proposed solutions and lessons learned from past public health events. The findings of the roundtable emphasized the importance of multisectoral coordination in addressing multifaceted public health events. Multisectoral coordination was described as the “master key” for tackling complex issues at the human-animal-environmental interface. The functionality of coordination within national structures during peacetime was deemed essential for its operationality during emergencies. Therefore, panelists recommended adopting a systematic approach to emergency coordination which includes identifying sector leaders, identifying the main coordination activities, exercising plans and continuous quality improvement. Additionally, the purpose of coordination should be clearly identified and articulated alongside the shared benefits for improved engagement of relevant sectors. Several challenges to effective coordination were identified, along with corresponding solutions. These included limited operational effectiveness of coordination mechanisms during peacetime, lack of awareness regarding the importance of coordination, limited trust and co-ownership within and between organizations, and competing priorities and conflicts of interest.

## Introduction

Public health events are complex, multifaceted and cannot be addressed effectively by one sector alone. Involvement of relevant sectors in the planning, preparedness, response, and recovery phases of an emergency or public health event is essential for mitigating risks, as well as securing national, regional, and global health security. A holistic, multidisciplinary, and multisectoral approach, involving a diverse range of disciplines, should be implemented in countries in compliance with the International Health Regulations (IHR) (2005) (1). Engagement of high-level policymakers, decision makers and communities are essential to enable a whole government and whole of society approach (2). In addition to the fact that multisectoral coordination is essential to address the complexity of emergency management, multisectoral coordination can be leveraged to address multiple issues, such as antimicrobial resistance, HIV/AIDS prevention, malaria elimination, tobacco control, climate change and NCD-related events. Therefore, an institutionalized multisectoral coordination mechanism can serve the Eastern Mediterranean Region (EMR) in multiple ways.

The concept of multisectoral coordination has been comprehensively described in multiple frameworks, such as the “Multisectoral preparedness coordination framework” (3) and “Taking a multisectoral, One Health approach: a quadripartite guide to addressing zoonotic diseases in countries” (4). Additionally, multisectoral coordination was mentioned as an overarching principle in the “Global Action Plan for the prevention and control of NCDs, 2013–2020” (5) and is recognized as one of the core principles of Primary Health Care, which is essential for achieving Universal Health Coverage. However, the institutionalization of the concept is highly complex and requires further exploration, keeping in mind countries’ diverse contexts in which multisectoral coordination is implemented.

The EMR has a population of more than 600 million people and faces several challenges, including health and humanitarian emergencies on an unprecedented scale (6). The EMR struggles with political and economic instability, weakened health systems, insufficient partners with strong operational presence, lack of trust in government, and poor multisectoral coordination (2, 7, 8).

The objective of this manuscript is to present key insights from the roundtable session titled “Strengthening Multisectoral Coordination and Capacity: From Strategies to Actions,” held during the 8th Biennial Regional Conference of the Eastern Mediterranean Public Health Network (EMPHNET), from September 15–18, 2024. The session highlighted the critical role of multisectoral coordination in public health, explored strategies for formalizing and implementing multisectoral coordination structures, and shared best practices, challenges, proposed solutions, and lessons learned from various initiatives aimed at strengthening multisectoral coordination.

## Roundtable description

The roundtable provided a platform for global and regional experts in multisectoral coordination to share knowledge, expertise, and best practices regarding multisectoral coordination implementation and formalization within national structures, particularly in the EMR. Discussions also addressed the challenges associated with effective multisectoral coordination implementation and sustainability, drawing on One Health experiences and lessons learned from past pandemics, epidemics and public health events.

The two-hour roundtable brought together five distinguished panelists, each contributing unique insights and expertise to the discussion on multisectoral coordination in public health, along with valuable input from the audience. The session began with a discussion on the definition and importance of multisectoral coordination, and its scope in public health. Professor Mahmudur Rahman, EMPHNET’s Country Director for the Bangladesh Office, and Dr. Mohammed Youbi, Director of the Directorate of Epidemiology and Disease Control at the Ministry of Health in Morocco, shared valuable lessons on the institutionalization and operationalization of multisectoral coordination, along with their experiences overseeing One Health projects. Dr. Scott Dowell, Senior Advisor at the World Health Organization for the Global Health Emergency Corps, provided insights on when and how to engage relevant sectors in multisectoral coordination efforts. Dr. Muhammad Sartaj, Regional Lead for the Eastern Mediterranean Region and Country Lead for the UKHSA International Health Regulation Strengthening Project in Pakistan, Dr. Jonathan Suk, Principal Expert at the Emergency Preparedness and Response, at the European Centre for Disease Prevention and Control (ECDC) and Dr. Heba Mahrous, a One Health Technical Officer at the World Health Organization Regional Office for the Eastern Mediterranean (WHO-EMRO), offered regional perspectives, highlighting lessons learned, challenges, and barriers to effective multisectoral coordination initiatives.

The roundtable engaged 67 public health professionals from various countries and organizations, including ministerial officials, policy/decision makers, IHR focal points, One Health experts, Field Epidemiology Training Programs (FETP) residents and graduates, and Rapid Response Teams (RRTs) members.

The roundtable discussions were centered around four main topics, (1) the concept of multisectoral coordination, (2) formalization of multisectoral coordination within governmental bodies, (3) best practices and lessons learned in implementing multisectoral coordination and (4) the challenges that hinder effective multisectoral coordination efforts, along with proposed solutions. These four topics were pre-identified to streamline and focus the discussion. Below is a narrative synthesis of the discussion.

## The concept of multisectoral coordination

Multisectoral coordination is recognized as crucial for the success of public health programs. In fact, a One Health approach is seen as essential for maintaining health security at the national, regional, and global levels (9). Furthermore, health risks are not solely the responsibility of the health sector, as many public health issues are linked to non-health sectors. Consequently, effective control measures may also need to involve these sectors, along with the private sector and the community. Despite the importance of coordination, there remains a level of ambiguity regarding its’ definition and how it differs from related terms such as collaboration and cooperation. In literature these terms are often used interchangeably, leading to overlapping definitions that lack clarity and cause confusion or uncertainty among public health practitioners (10).

Panelists defined coordination as the organization and management of human and/or physical resources to achieve a common goal, recognizing it as a core element of collaboration. On the other hand, collaboration was described as an evolving process involving the active and reciprocal engagement of two or more social entities (e.g., people, teams, organizations) working together toward a shared goal. Collaboration also occurs at multiple levels, including within the same team, across teams, departments, organizations, sectors, and even between countries. While coordination focuses on organizing people, organizations, or resources, it does not always require reciprocal engagement. For example, a team leader delegating tasks to team members demonstrates coordination, as does the allocation of vaccines from national warehouses to subnational storage centers. Collaboration, however, inherently involves active engagement of social entities as a fundamental component of its definition.

The WHO, in its global action plan for health lives and well-being for all, highlighted that successful collaboration includes coordinated actions among diverse and pertinent stakeholders (11). Dr. Sartaj illustrated the difference between coordination and collaboration with the example of an orchestra. “*During a performance, the conductor coordinates the musicians by guiding them with hand movements as they play a symphony. This is coordination. However, the process that takes place before the performance—such as rehearsals, sitting together, and sharing knowledge—is an example of collaboration*.”

This theme was concluded with an example that reiterated the difference between coordination and collaboration, referencing EMPHNET’s conference. *“I think what EMPHNET is doing today in terms of organizing this event, is coordination. But what they have done over the years in bringing and working with all partners and developing the Field Epidemiology Training Program (FETP) is the collaboration leading to this event. So, I think that will be my view on coordination and collaboration.” Dr Sartaj.*

## Formalizing multisectoral coordination within governmental bodies

The integration of coordination mechanisms and procedures within governmental bodies requires the establishment of supportive policies, legislation, and frameworks to enable their effective operationalization. Enacting such legal frameworks strengthens the national governance of coordination bodies, facilitates the allocation and mobilization of resources during both peacetime and emergencies, and enhances accountability among all involved stakeholders.

Furthermore, the purpose and rationale for engaging sectors must be clearly delineated and articulated to ensure the right sectors are involved, optimize resource use, increase transparency, and foster trust among partners. Since not all public health issues require multisectoral coordination, it is crucial to identify roles and responsibilities from the outset. Identifying relevant sectors based on priority indicators is essential. EMPHNET’s Stakeholder Mapping and Analysis Tool (12) uses three main domains to assess and prioritize stakeholders in relation to the project or initiative. These domains are as follows: Expertise (Contribution and Legitimacy), Willingness to engage and Value (Influence, Necessity of Involvement and Extent of geographical involvement). Mapping stakeholders is only the first step. To truly engage relevant sectors, gain their buy-in, and advocate for a multisectoral approach, it is crucial to emphasize shared benefits, especially since conflicts of interest, including competing priorities among sectors, may arise. Following the identification of the sectors, and formulation of the coordination mechanism, Dr. Dowell recommends that countries employ a systematic approach to public health emergency coordination. This approach considers continuous quality improvement and includes four main steps:

1. Identifying the leaders: Three to five leaders from relevant sectors should be identified in advance using a stakeholder mapping tool, with selection guided by the objectives of the planned initiative.
2. Identify the main coordination activities: The leaders will agree on the planned activities and take responsibility for coordinating their implementation throughout the emergency cycle—prevention, preparedness, response, and recovery. They will also serve as key communicators with the public and decision-makers during public health emergencies.
3. Practice: Coordination plans should be jointly developed, co-owned and practiced through simulation exercises that include scenarios not typically encountered in routine practice.
4. Use a checklist: Implementing a checklist is a way to ensure that activities are carried out according to plans and best practices. A checklist will help leaders identify gaps and challenges that need to be addressed for improved preparedness and response, and it can also enhance documentation related to these processes. Such checklists should also be jointly developed and co-owned by sector leaders.

To ensure the sustainability of public health initiatives, the effective prioritization of health issues and strategies, and the avoidance of duplicative efforts, partners and funders must invest in strengthening national systems by collaborating and coordinating with authoritative, influential national officials who hold decision-making power. Additionally, they should avoid creating operational silos, as these can lead to further fragmentation, inequities, and health disparities across the population.

## Best practices and lessons learned in implementing multisectoral coordination

This theme began with reflections and lessons learned from the preparedness and response to the COVID-19 pandemic. Dr. Dowell stated that *“…multisectoral coordination was critical for Covid and Covid was the most important test of multisectoral coordination.…And most countries failed.”* The COVID-19 pandemic has massively impacted global economy, public health, and disrupted education (13) as well as travel and trade (14). The collective efforts of the WHO, scientists, researchers, governments, health authorities, and Non-Governmental Organizations (NGOs), mitigated some of the health, economic and societal consequences of the pandemic. Such collaboration enhanced the knowledge of the disease’s etiology, origin, epidemiology, and prevention and treatment measures (13). Panelists agreed that countries with well-integrated coordination mechanisms and pre-established relationships between sectors had the upper hand in responding to the pandemic. They were able to quickly mobilize decision making communities related to education, continuation of the health sector and the influence of the media.

Media plays a significant role during public health emergencies. Dr. Rahman stated, “*I think media partner is very important in every aspect, for health promotion, prevention, and also mitigation aspect during emergencies*.” However, if contradictory or inaccurate information is disseminated it could potentially distort reality, and instill irrational fear among the population, as well as cause societal tension and mistrust in the government. Media should use its influence to enhance response efforts, minimize misinformation and prevent the harmful consequences of disinformation. Morocco’s experience during COVID-19 was highlighted to showcase best practices in media engagement and public communication. Dr. Youbi stated *“During Covid-19, we decided in Morocco to be fully transparent, and to share real-time information with the media, about the number of cases, case fatality rates, actions taken, and what is needed from the public. And that worked very very well.”* It is critical that the media understand its role and influence in public health, particularly surveillance related activities and risk management, such as awareness raising and risk communication. The media can be a key player in surveillance and serve as an importance source of information for Event Based Surveillance.

Panelists agreed that for effective preparedness and response, as well as for a well-functioning and integrated multisectoral structure during emergencies, it must also be operational in peacetime. As Dr. Mahrous stated, “*Sustaining multisectoral initiatives, such as RRT, during peacetime demonstrates that the RRT is well integrated into the broader emergency response framework*.”

Panelists agreed that public health events whether minor or large scale, should be used as lessons to prepare for future occurrences. The utilization of evaluation methods such as simulation exercises (tabletop exercises and drills), and After Action Reviews (AAR) is the cornerstone of improved preparedness and enhanced multisectoral and multidisciplinary coordination. Such exercises enable sectors to reach consensus on roles, responsibilities and best practices, while also building both individual and institutional capacity. The recommendations and findings from these exercises should be integrated within plans, Standard Operating Procedures (SOPs) and national systems. Dr. Suk noted the emphasis that ECDC places on cross-sectoral coordination, and that One Health related topics are a strong example of this. The EU Health Security Initiative (15) has discussed this in detail with countries in the region, during a recent meeting on climate change and infectious disease organized together with Egypt (16). Dr. Suk reflected on the ECDC’s experience in addressing the increased number of human cases of West Nile Fever that occurred across Europe during 2018. This rise represented a 7.2-fold increase from the previous year (17). Dr. Suk, mentioned that a series of after action reviews were conducted that involved affected EU countries and neighboring nations. Discussions were centered around response actions, challenges, and areas for improvement. Actions were analyzed and reviewed, and recommendations drafted, and plans were revisited. Dr. Suk mentioned *“…I think that in all those countries, after those reviews, they were able to adopt and revise not just the generic broader One Health plans, but their specific response plans for mosquito borne diseases for West Nile Virus (WNV), which is very, quite productive.”* The cross-country review meeting strengthened cross-border collaboration and allowed the sharing of best practices and experiences. Dr. Dowell emphasized the importance of cross-border collaboration, “It may not matter if we do a good job in our country about collaboration, if we do not have a way to collaborate with the neighboring countries and countries distant.”

Despite several success stories and lessons learned, panelists realized that there remains multiple challenges and barriers hindering an effective coordination during public health emergencies.

## Challenges that hinder multisectoral coordination and their proposed solutions

Panelists realized the challenges of addressing multifaceted public health issues. Dr. Mahrous described multisectoral coordination as a master key of such issues; *“I consider multisectoral coordination to be a “master key” that reliably unlocks the* var*ious locks we face—namely, our health challenges. The application of a One Health approach is particularly crucial at tackling the threats at the human-animal-environment interface.”* This is especially important for a region like the EMR, where the current political landscape is marked by protracted emergencies, ongoing conflicts, political instability, displacement, resource limitations, sanctions, and the added complexities of climate change (18–20). These challenges underscore the critical need for multisectoral collaboration and coordination, especially during times of hardships and resource scarcity.

Additional challenges of effective multisectoral coordination raised during the roundtable included the limited operational effectiveness of coordination mechanisms during peacetime, in contrast to their utility during emergencies, lack of awareness regarding the importance of coordination, limited trust and co-ownership within and between organizations, and competing priorities and conflicts of interest. Table 1 highlights the challenges and their proposed solutions.

**Table 1:** Challenges of multisectoral coordination and proposed solutions.

Challenges	Solutions
Governance	
Competing priorities among different sectors (21–23).	Key stakeholders should showcase the shared benefits of public health initiatives and foster trust among the stakeholders.
Lack of accountability mechanisms	Stakeholders should secure high-level political commitment to ensure a strong foundation for action and to reinforce accountability across sectors (21). This can be achieved by advocating to decision-makers, senior government officials, and funders. Establish a robust accountability mechanism and a comprehensive monitoring and evaluation framework to track the implementation of planned activities and ensure stakeholder accountability.
Lack of clear leadership and supporting policies (24–26)	Strengthen national governance and leadership in coordination mechanisms by advocating for and/or developing, implementing, and endorsing supportive coordination policies and regulations. Leadership can be agreed among partners based on a rotatory basis or on the threat/areas of concern.
Trust	
Conflicts of interest	Promote transparency and open dialogue.
Limited trust and co-ownership within and between organizations	Articulate the roles and responsibilities of the different organizations through jointly developed action plans that incorporate accountability mechanisms, such as regular meetings, specific deliverables and evaluation indicators. Engage partners and relevant sectors early on and communicate the shared benefits of the initiative.
Communication	
Lack of communication and relationships between sectors (27).	Developing and maintaining an information sharing platform among sectors to foster dialogue, knowledge exchange and transparency Stakeholders within the coordination platform should hold regular meetings to ensure effective collaboration and information sharing (28).
Countering misinformation and disinformation (8)	Media is a key partner and should be engaged in the planning and response phases of emergencies. Transparent communication between the media and other sectors (28). Effective risk communication, that is timely, transparent and culturally appropriate.
Limited awareness regarding the importance of coordination	Communicate transparently the shared benefits and evidence-based information of following a multisectoral approach (29).
*Ad hoc* and unsustainable efforts (8).	A dedicated venue for structured discussion ensures ongoing multisectoral collaboration (30)
Capacity building	
Limited capacity building efforts, particularly soft skills (25).	Organizations should formalize capacity building efforts, including soft skills as part of their structure (33).
Resource Limitations (31, 32)	Mobilizing and coordinating investments from development partners, research institutions, and the private sector (24).

## Conclusion

Multisectoral coordination is essential for national, regional and global health security. In regions characterized by political instability, scarce resources and limited funding, such as the EMR, effective coordination is necessary to optimize resource mobilization and enhance overall public health response. Coordination should be formalized within national structures, and operational during peacetime and emergencies.

## Recommendations

1. Strengthen multisectoral and multidisciplinary coordination efforts, across the health and non-health sector, including the private sector and communities to build a resilient health system.
2. Institutionalize coordination mechanisms within organizations and ensure their operational effectiveness during peacetime for effective preparedness and response.
3. Establish transparent, two-way communication channels with the media to strengthen public communication, build community trust, and effectively counter misinformation and disinformation during emergencies
4. Conduct regular simulation exercises—including tabletop exercises and drills—to test multisectoral plans and SOPs. These exercises should aim to build capacity, clarify roles and responsibilities, identify gaps, and inform the revision of plans
5. Conduct AARs immediately following acute public health events to assess actual responses against intended actions, identify challenges and best practices, and revise plans accordingly.

## Data Availability

All data generated or analyzed in this study are included in the article. Further inquiries can be directed to the corresponding authors.
